# Physical and Psychological Impact of the Phase One Lockdown for COVID-19 on Italians

**DOI:** 10.3389/fpsyg.2020.563722

**Published:** 2020-12-17

**Authors:** Marco Tommasi, Francesca Toro, Simone Arnò, Angelo Carrieri, Marco Maria Conte, Marianna Daria Devastato, Laura Picconi, Maria Rita Sergi, Aristide Saggino

**Affiliations:** Department of Medicine and Aging Science, University of Chieti-Pescara, Chieti, Italy

**Keywords:** COVID-19, anxiety, depression, resilience, lockdown

## Abstract

The exceptional pandemic due to the 2019 coronavirus disease (COVID-19) has obliged all Italians to stay at home. In the literature, there are evidences that traumatic global events, such as natural catastrophes and pandemic, have negative effects on the physical and psychological health of the population. We carried out a survey to analyze the physical and psychological conditions of Italians during the pandemic. Due to the severe limitations in moving during the phase one lockdown, the survey was administered by internet. Results show that Italians followed the provisions established by the Italian government to avoid contamination, but 43% of them declared to have suffered from physical symptoms, in particular migraine, sleep disorders, persistent exhaustion, and difficulty of concentration. They have great fear to be contaminated or that relatives or friends can be contaminated, and they actively take actions to avoid contamination. Participants declared that they had suffered a lot of inconveniences due to restrictions in their movements, and that their life habits were strongly changed. They spent their time at home in different activities, but their psychological well-being was strongly impaired by the lockdown. The level of anxiety tripled, in relation to the prepandemic period, and 30% of males and 41% of females declared to have severe levels of depression. Participants with high levels of optimism and hopefulness show a stronger resilience against anxiety and depression. In addition, there is a negative correlation between anxiety and depression and the five factors of personality. These results show that psychological diseases must not be neglected, and that people in lockdown do need support for their psychological health, also with the help of internet and communication technologies.

## Introduction

The 2019 coronavirus disease (COVID-19) pandemic is a global event that is causing enormous changes in lifestyles and daily activities of people of every part of the world. In Italy, particularly, the pandemic has caused a high level of deaths among Italian citizens (14.1% of the contaminated population), especially among persons who are over 65 years old. In all the world, there are more than 4.5 million cases of contaminated people with more than 312,000 deaths [data obtained from the Center for Systems Science and Engineering (CSSE) at Johns Hopkins University on 5 May 2020^1^). On the basis of this high risk for citizens health, the Italian government from 10 March declared the lockdown for all working and social activities (phase one lockdown); the closure of schools, universities, public offices, and private businesses; and the mandatory quarantine for all Italians who were contaminated by the virus. Italians have to stay at home, with the possibility to move outside only in cases of strong necessity, and they have to follow procedures for securing themselves against contamination. In addition, the Italian government reinforced the public medical system to deal with the pandemic. We decided to analyze, with a survey, the impact of the pandemic and of the first phase of the lockdown on the behavior and psychological well-being of people.

There are evidences from the literature that global negative events, such as natural catastrophes, cause physical damages and psychological distress (Janney et al., 1977; Kanno et al., 2013). Also, the pandemic has negative effects on psychological well-being, not only in physicians and medical workers who have to deal with the effects of the pandemic on human beings (Greenberg et al., 2020; Tan et al., 2020) but also in normal people who have to abruptly change their life habits (Wheaton et al., 2012; Sood, 2020). COVID-19 immediately emerged as a dangerous virus for human beings health because of its high level of contamination, and researchers recommended an immediate intervention to reduce its dangerousness (Wang et al., 2020). By reviewing studies of the psychological impact of previous pandemics [e.g., severe acute respiratory syndrome (SARS), H1N1, or Ebola], Brooks et al. (2020) evidenced that during the pandemic period, people suffer from stress, depression, and anxiety and can develop fears and worries about their economic status. The authors also claim that further research is necessary to analyze the impact of public health measures activated for preventing contamination and the real efficiency of these measures (Brooks et al., 2020). The pandemic can have negative impacts on physical health (Li et al., 2020), but other authors highlighted the strong necessity to study the effects of the pandemic and government restrictions of individual activities on people’s psychological health, especially on their level of anxiety and depression (Holmes et al., 2020). Psychological stress, anxiety, and depression have negative impacts not only on human beings but also on the entire society, from both an economic and a political standpoint (Gyani et al., 2013; Layard, 2013).

Italy was the first European country to face the risk of a pandemic on a large scale (Saglietto et al., 2020). The rapid increase of positive cases in the last days of February induced the Italian government to take severe measures that blocked nearly all working and social activities, and these measures could have had a strong impact on Italians’ mental health. In particular, the duration of the restrictive measures against free movement, the reduction of social contacts, the fear of possible infections, the shortage of economic resources or supplies, and the lack of clarity in information could have negatively affected citizens and their psychological well-being (Orrù et al., 2020); thus, methods of prevention for stress and mental health diseases (Marazziti et al., 2020) should be taken into consideration, also by the public authority, to reduce pandemic distress. Therefore, the pandemic can be considered a strong cause of stress for Italian people without any doubts, and it is necessary to estimate the level of psychological disease generated by the pandemic in order to develop the best methods of interventions to contrast its harmful impact on the life of each individual and on the functioning of the entire society. However, empirical data are necessary to evaluate the real physical and psychological conditions of the population.

## Materials and Methods

### Sample

The sample was composed of 418 participants (72.97% females). Mean age was 32.23 years (SD = 12.46). Table 1 shows the demographic characteristics of the sample. Even if there were participants of nearly all Italian regions (only Valle d’Aosta was not present), a higher percentage of participants were from the central and southern parts of Italy.

**TABLE 1:** Demographic characteristics and distribution of the Italian sample.

**Italian regions**	**%**	**Professions**	**%**	**Marital status**	**%**
Abruzzo	42.82	Peasant, farmer, or fisherman	0.48	Divorced or separated	3.11
Basilicata	1.91	Driver	0.96	Engaged or cohabitant	34.69
Calabria	6.70	Unemployed	8.13	Single	36.84
Campania	9.81	Retailer or shop keeper	2.15	Married	24.88
Emilia-Romagna	1.67	Employee	4.55	Widowed	0.48
Friuli Venezia Giulia	0.48	Teacher or professor	9.57		
				
Lazio	4.07	Dependent worker	17.46	**Education level**	**%**
				
Liguria	0.24	Seasonal worker	0.96	Primary school	0.72
Lombardia	4.07	Not qualified worker	0.96	Secondary school	5.74
Marche	3.59	Manager or businessman	2.39	High school	53.35
Molise	1.67	Military worker	0.72	University	4.19
Piemonte	0.48	Pensioner	1.91		
				
Puglia	15.79	Self-employed worker	9.33	**Annual familiar income (**€)	**%**
				
Sardegna	0.72	Student	39.47	<20,000	44.02
Sicilia	0.72	Technician or qualified worker	0.96	From 20,000 to 40,000	38.52
Toscana	2.39			From 40,000 to 60,000	1.29
Trentino-Alto Adige	0.48			From 60,000 to 80,000	2.87
Umbria	0.72			>80,000	4.31
Veneto	0.48				
Missing	1.20				

### Materials

The online survey included different questions and psychological questionnaires to collect data about the physical and psychological conditions and the diseases produced by quarantine on participants. In addition, we provided questions about the level of knowledge of COVID-19 and its mechanisms of propagations. Personality traits, anxiety, depression, and resilience of participants were measured with standardized psychological tests.

### Assessment of Participants’ Health Status

In the survey, questions were provided to collect information about the health status of participants. We asked if participants were contaminated or believed to have been contaminated by COVID-19, if they were eventually recovered in hospitals, if they suffered from other chronic pathologies (and in case of positive response, which was their pathology), what kind of physical symptoms they suffered in the last 2 weeks, how long was the duration of these symptoms, and if they had relatives or friends recovered from COVID-19.

### Assessment of Participants’ Knowledge and Fear About COVID-19

To assess participants’ knowledge of COVID-19, a series of questions was used asking participants if they had correct or incorrect notions about COVID-19. For example, a correct notion is that COVID-19 causes respiratory diseases, and an incorrect notion is that it can infect only old people. Participants responded using a Likert scale from 1 (not true) to 4 (totally true). Another series of questions assessed participants’ knowledge of the various means of transmission of COVID-19 (e.g., *via* physical contact or fluid contamination). Participants had to evaluate the most probable means of contamination using a Likert scale from 1 (not true) to 4 (totally true). Other questions assessed different kinds of fear about COVID-19 (e.g., to be contaminated or that relatives or friends can be contaminated) using a Likert scale from 1 (no fear) to 4 (highest fear).

### Assessment of Participants’ Problems and Behavior During the Lockdown

Participants were asked which were the most important problems they suffered after the movement restrictions due to lockdown or quarantine (e.g., obligation to stay at home or possible economic difficulties) using a Likert scale from 1 (not true) to 4 (totally true). We asked participants to rate the global disease caused by the lockdown (no disease, low disease, medium disease, high disease) and how strong was the impact of the lockdown on their life habits (no impact, low impact, medium impact, high impact, total impact). To assess the actions or behaviors participants took on to contrast contamination, we asked if they remained at home and what they did to avoid infection (e.g., if they washed their hands or wore masks when going out). In addition, we asked what kind of activity they were doing during their permanence at home when they were not working (e.g., watching TV or reading books). Other questions were provided to assess if they received social support from family, local institutions, or voluntary associations during the lockdown (“Are you receiving any help from someone or from services and institutions?”).

### Assessment of Psychological Conditions

We assessed the personality traits of the five factor model of personality (extraversion, agreeableness, conscientiousness, emotional stability, and openness) using the Big Five Observer (BFO) questionnaire (Caprara et al., 1994). The BFO is composed of 40 items that are a couple of adjectives that define the characteristic of the five traits of personality. Scores were based on a Likert scale from 1 to 7. The higher the score, the higher the presence of the trait. Emotional stability of the BFO is the inverted measure of neuroticism (Caprara et al., 1994).

We assessed anxiety and depression of participants to have indications of their psychological well-being during the lockdown period. Anxiety was measured using the State Trait Anxiety Inventory (STAI) of Spielberger with 20 items (Spielberger, 1983). Scores were based on a Likert scale from 1 (not at all) to 4 (very much). We used the standardized scores of the Italian population collected for the CBA 2.0 to estimate if participants suffered from a severe level of anxiety (Sanavio, 1997). The global STAI scores corresponding to the 95th percentile were 55 and 61 for males and females, respectively. Depression was assessed using the short form of the Beck Depression Inventory (BDI) with 13 items (Beck and Beck, 1972). The Italian version of the BDI was validated by Sica and Ghisi (2007). Scores ranged from 0 to 3 for each item. Different levels of depression severity were established in relation to range scores (Beck and Beck, 1972; Reynolds and Gould, 1981; Knight, 1984; Stukenberg et al., 1990). Scores from 0 to 4 indicate the absence of depression, from 5 to 7 mild depression, from 8 to 15 moderate depression, and scores higher than 15 indicate severe depression. Participants’ resilience was estimated using the Revised Life Orientation Test (LOT-R) scale by Scheier et al. (1994), which measures optimism, and the Hope Herth Index (HHI) scale (Herth, 1992), which measures hopefulness. Resilience is composed of many characteristics or qualities (Richardson, 2002), and among the optimal characteristics indicated in a special issue of the American Psychologist and of the Journal of Social and Clinical Psychology, there are optimism (Peterson, 2000) and hope (Snyder, 2000). We selected optimism and hopefulness because other studies defined them as protective factors against traumas and negative life events (Madsen and Abell, 2010). The LOT-R scale has 10 items, with scores on a Likert scale from 1 (strongly disagree) to 5 (strongly agree), and it measures the level of optimism and faith on positive outcomes in the future. The higher the score, the higher the level of optimism. The Italian standardization of the LOT-R was made by Chiesi et al. (2013). The Italian standardization of the HHI was made by Ripamonti et al. (2012). The scale consists of 12 items with a Likert scale from 1 (strongly disagree) to 4 (strongly agree), and it assesses three dimensions of hopefulness: inner sense of temporality and future, that is the ability to preserve a positive vision of the future (HHI temp); interconnectedness with self and others or subjective beliefs to have a strong interior force and to be not isolated from others (HHI conn); and inner positive readiness and expectancy, that is the ability to react to negative situations and the confidence that our personal actions can improve negative situations (HHI exp). This scale was used particularly for estimating resilience in patients suffering from cancer disease (Ripamonti et al., 2012). In addition, we used the Italian standardized version of the Marlowe–Crowne (MC) scale for social desirability (Manganelli Rattazzi et al., 2000), to check the validity of subjective responses in psychological scales. We used the short form with nine items with a Likert scale from 1 (low social desirability) to 5 (high social desirability). Positive or negative correlations with MC indicate a tendency to over- or underestimate psychological characteristics or traits, respectively.

### Procedure

Participants responded by compiling an online survey made with Google modules. The link of the survey was distributed *via* social networks (Facebook and WhatsApp). Participants were contacted through the social networks of the authors of this work and were asked to propagate the link to other relatives or friends. Participants before doing the survey were informed about the aims of the research, and they were given information about the privacy of their data. We followed the Helsinki Declaration of ethical principles for medical research involving human participants, and the study procedure received the approbation of the Department of Medicine and Aging Sciences for its execution. Before compiling the survey, participants had to declare their effective will to participate in the survey. Without this declaration, they could not start the compilation. Participation was voluntary. Because of the possibility to repeat the test, at the end of the survey, we asked participants if they wanted to compile the survey on a successive moment. Data were collected from 8 April to 5 May. Anonymity and privacy of the participants were guaranteed according the Italian and the European laws about privacy (Italian law n. 196/2003 and EU GDPR 679/2016, respectively).

## Results

### Missing Data

The online survey was projected to reduce the risk of missing data. For completing the survey, it was necessary to respond to all items. In addition, some items were activated only when participants declared to possess specific characteristics (e.g., items asking the kind of chronic pathology, if the participant declared it). In this way, we reduced the number of missing data to zero.

### Assessment of Participants’ Health Status

Table 2 shows the participant’s health status. Nearly all participants declared not to be contaminated by COVID-19 (>95%). Of the participants, 4% declared that they believed to be contaminated, even if they did not make any medical test, and less than 1% declared to have been effectively contaminated.

**TABLE 2:** Participants’ health status.

**Do you think to be contaminated by COVID-19?**	**%**
No	95.22
Yes, but I did not make any medical test	4.07
Yes, I made the medical test	0.72

**Have you been recovered in a hospital for COVID-19?**	**%**

No	98.99
Yes	1.01

**Do you suffer from chronic pathologies (different from COVID-19)?**	**%**

No	91.87
Yes	8.13

**Other patologies**	**%**

Asthma or respiratory disease	19.45
Diabetes	16.68
Inflammatory diseases	13.90
Migraine	11.12
Hypertension	11.11
Thyroid disease	8.34
Cancer	5.56
Renal impairment	5.56
Multiple sclerosis	2.78
Not specified	5.56

**Did you suffered from some physical symptoms in the last 2 weeks?**	**%**

No	56.94
Yes	43.06

**Symptoms duration**	**%**

From 1 to 3 days	39.18
From 3 to 5 days	23.71
From 5 to 10 days	16.49
More than 10 days	2.62

**Did you have relative or friends infected by COVID-19?**	**%**

No	72.01
Yes	20.10
Do not know	7.89

**FIGURE 1:**
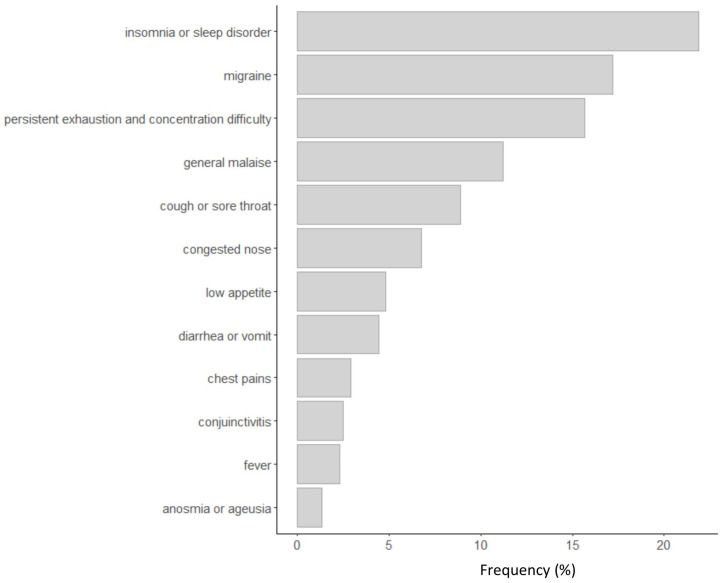
Frequency of physical symptoms declared by participants in the last 2 weeks.

Only 1% of participants reported to have been hospitalized for COVID-19. Furthermore, 8.13% of participants suffered from chronic diseases, not related to COVID-19. The most frequent diseases were asthma, diabetes, inflammatory diseases, migraine, hypertension, and thyroid disease. In addition, 43% of participants declared to have suffered from physical symptoms in the last 2 weeks. The majority of symptoms lasted less than 10 days. Moreover, 20.1% of participants declared that they had some relatives or friends infected by COVID-19. Figure 1 shows the frequency of the different symptoms declared by participants. The most prevalent physical symptoms were insomnia or sleep disorder, followed by migraine, persistent exhaustion, and general malaise.

### Assessment of Participants’ Knowledge and Fear About COVID-19

Table 3 shows the mean ratings and standard deviation of participants’ scores when they were asked to respond to some items to assess their knowledge and fear about COVID-19 and the risk to be contaminated.

**TABLE 3:** Mean ratings and standard deviation (SD) of participants’ scores to assess knowledge and fear about COVID-19 and its means of contamination.

**Covid knowledge**		**Covid fear**		**Covid means of contamination**	
**Items**	**Means (SD)**	**Items**	**Means (SD)**	**Items**	**Means (SD)**
It causes respiratory diseases	3.56 (0.56)	To get seriously ill	2.92 (0.91)	By contact with fluids(e.g., blood)	2.84 (1.03)
It was generated in a laboratory	1.82 (0.89)	My beloved persons got harmed by the virus	3.62 (0.54)	By cough or sneeze	3.67 (0.49)
It was created by a secret agency	1.34 (0.65)	Damages in my profession or work	2.37 (0.98)	By contact with objects or clothings	2.66 (0.86)
It is a banal flu	1.37 (0.61)	Generation of wars or social conflicts	2.59 (0.93)	By air conditioning installations	2.07 (0.94)
It affects only old people	1.57 (0.76)	Impossibility to find a vaccine	2.48 (1.01)	By food	1.55 (0.78)
It can damage everyone	3.63 (0.54)	Impossibility to find valid therapies against the virus	2.58 (0.97)	By domestic animals	1.17 (0.43)
It can kill only people with other illnesses	2.12 (0.83)	The high probability to be contaminated	3.33 (0.71)	By wild animals	1.42 (0.70)
Animals transmitted it to human beings	2.44 (1.12)	High level of virus mutation	3.12 (0.84)		
It can be defeated only with medicines	2.20 (0.98)				

Participants know that COVID-19 creates respiratory disease, that it can contaminate everyone, and that, initially, it was transmitted by animals to humans. Participants do not generally believe that COVID-19 is an artificial virus created in the laboratory, that it is only a banal flu, and that it affects specifically old people. Participants show a high level of fear about COVID-19. The greatest fears are the possibility that beloved persons can be contaminated by the virus, the possibility to be contaminated, the genetic mutation of the virus, the difficulty to find valid therapies or vaccines, and the risk of social conflicts or wars. In relation to the means of contamination, participants say that the principal means are through air (cough or sneeze) and through contact with organic fluids (e.g., blood) or with contaminated objects.

### Assessment of Participants’ Problems and Behavior During the Lockdown

Table 4 shows what are the principal problems that caused disease in participants. The principal causes are the impossibility to see relatives or friends, the duration of the lockdown, and the obligation to stay at home.

**TABLE 4:** Lockdown disease causes, global level of disease, impact of phase one lockdown on normal life, and permanence at home of the sample.

**Quarantine disease causes**	**Means (SD)**
Obligation to stay at home	2.84 (0.93)
Impossibility to see relatives and friends	3.36 (0.71)
Impossibility to work	2.48 (1.04)
Reduced physical or sporting activity	2.29 (1.02)
Impossibility to receive adequate health care	2.08 (1.04)
Economic difficulties	2.22 (1.07)
Impossibility to attend schools for children	1.69 (0.95)
Long duration of lockdown	3.26 (0.83)

**General disease**	**%**

No disease	5.02
Low disease	35.17
Medium disease	43.30
High disease	16.51

**Impact of lockdown on life habits**	**%**

No impact	1.20
Low impact	12.44
Medium impact	36.36
High impact	27.51
Total impact	22.49

**Permanence at home**	**%**

I stay always at home	27.03
I get out only for necessity	72.01
I get out as before	0.96

**FIGURE 2:**
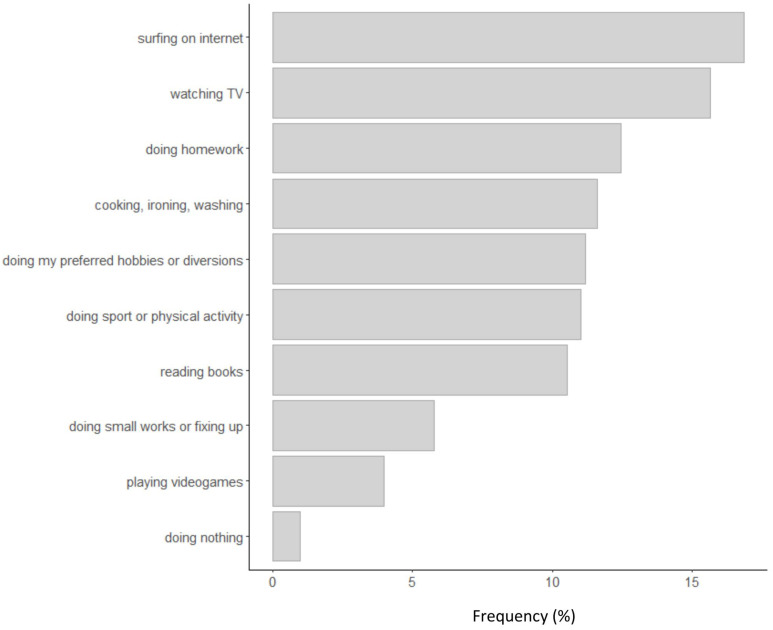
Frequency of activities practiced at home during the phase one lockdown.

Of the participants, 59.81% reported a medium or high level of general disease, and 50% declared that the quarantine strongly changed their life and habits. Furthermore, 27% of participants stayed always at home, whereas 72% got out home only for necessity. Less than 1% declared to get out as they did before the lockdown. Figure 2 shows the activities practiced at home during the lockdown (when not working) by participants.

**FIGURE 3:**
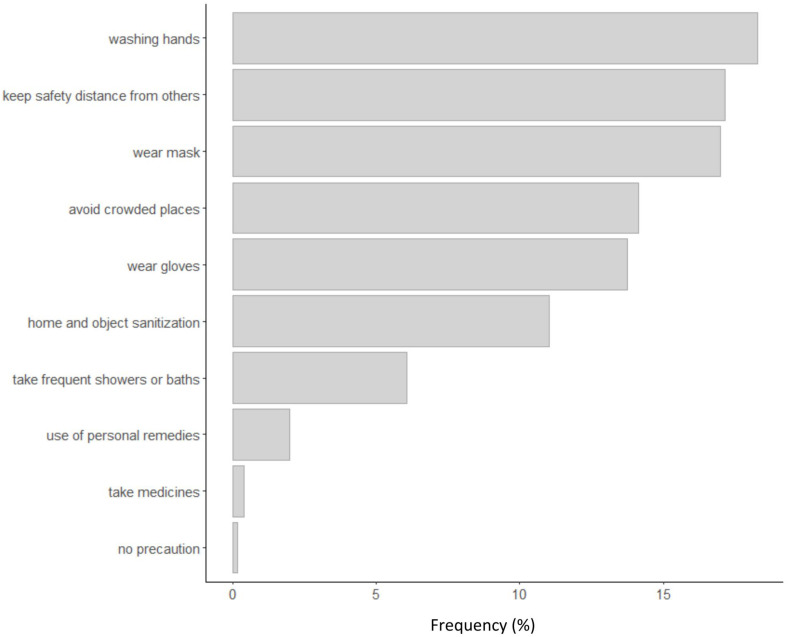
Frequency of precautions against contamination.

Many of them watched TV or used the internet and did homework or hobbies. Very few people were completely inactive (less than 1%). Figure 3 shows what were the most frequent precautions taken by participants to avoid contamination.

**FIGURE 4:**
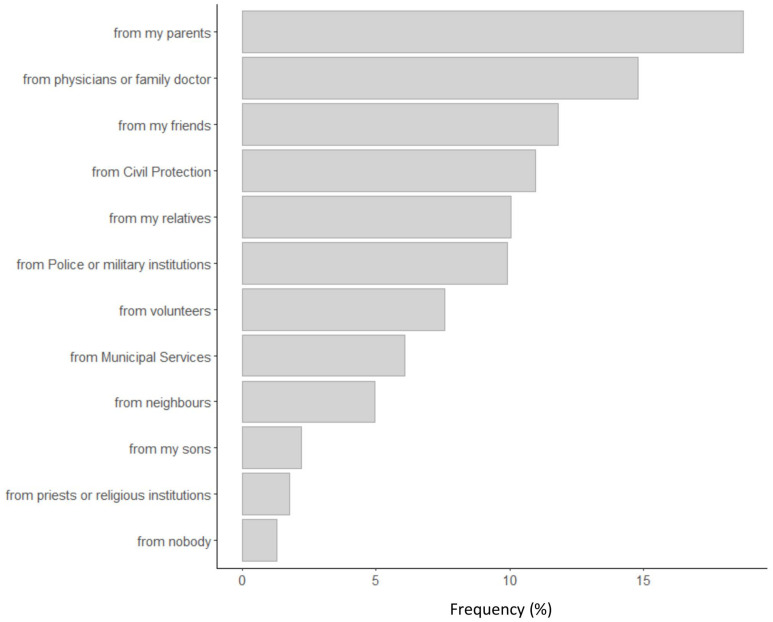
Frequency of social support agents for participants during the phase one lockdown.

The most frequent precautions were the use of masks and gloves, washing hands frequently, sanitization of objects and rooms, observance of a safety distance from others, and avoidance of crowded places. Therefore, participants followed the principal instructions of the Italian Ministry of Public Health to reduce contamination risks. Less than 0.2% of participants declared that they did not take any precaution against contamination. Figure 4 shows the principal agents of social support received by participants during the lockdown. Participants received social support principally from family (parents, sons, relatives), family doctors, friends, civil protection, police, and volunteers.

### Assessment of Psychological Conditions

Table 5 shows the descriptive, reliabilities and correlations of the psychological scales used to assess psychological well-being, psychological resilience, and personality. Values of skewness and kurtosis are included in the range of −2 and 2, confirming that score distributions are prevalently normal (Gravetter and Wallnau, 2014).

**TABLE 5:** Descriptive statistics (mean, standard deviation, skewness, and kurtosis), reliability (Cronbach’s α), and bivariate correlation between psychological variables and assessment of life change and disease generated by lockdown.

		**Life change**	**General disease**	**BFO-E**	**BFO-A**	**BFO-C**	**BFO-S**	**BFO-O**
	Mean	3.58	2.71	35.27	40.57	38.58	33.12	41.70
	Std. Dev.	1.01	0.80	7.22	5.90	6.56	8.34	6.20
	Skewness	−0.09	−0.04	−0.02	0.06	0.00	−0.13	0.38
	Kurtosis	0.12	0.12	0.37	−0.29	0.29	−0.23	−0.42
	Cronbach’s α	–	–	0.63	0.58	0.57	0.74	0.68
MC	Pearson’s *r*	−0.06	−0.07	0.17	0.47	0.32	0.33	0.19
	*p*-value	0.23	0.15	<0.001	<0.001	<0.001	<0.001	<0.001
STAI	Pearson’s *r*	0.19	0.42	−0.27	−0.39	−0.26	−0.68	−0.19
	*p*-value	<0.001	<0.001	<0.001	<0.001	<0.001	<0.001	<0.001
	Partial r*	0.16	0.41	−0.25	−0.38	−0.24	−0.66	−0.17
BDI	Pearson’s r	0.09	0.36	−0.38	−0.39	−0.39	−0.59	−0.25
	*p*-value	0.06	<0.001	<0.001	<0.001	<0.001	<0.001	<0.001
	Partial r*	0.07	0.34	−0.35	−0.36	−0.36	−0.56	−0.24

		**LOT-R**	**STAI**	**BDI**	**HHI temp**	**HHI conn**	**HHI exp**	**MC**

	Mean	19.53	45.78	13.39	11.89	11.93	12.90	30.40
	Std. Dev.	4.87	12.29	6.39	2.29	2.20	2.17	4.89
	Skewness	−0.22	0.33	0.32	−0.19	−0.48	−0.78	0.04
	Kurtosis	−0.22	−0.60	−0.05	−0.22	0.20	1.14	−0.19
	Cronbach’s α	0.82	0.94	0.78	0.68	0.57	0.71	0.64
MC	Pearson’s *r*	0.30	−0.26	−0.34	0.29	0.36	0.23	
	*p*-value	<0.001	<0.001	<0.001	<0.001	<0.001	<0.001	
STAI	Pearson’s *r*	−0.46	–	0.65	−0.59	−0.40	−0.39	
	*p*-value	<0.001	–	<0.001	<0.001	<0.001	<0.001	
	Partial r*	−0.45	–	0.63	−0.58	−0.39	−0.39	
BDI	Pearson’s *r*	−0.50	–	–	−0.64	−0.53	−0.54	
	*p*-value	<0.001	–	–	<0.001	<0.001	<0.001	
	Partial r*	−0.47	–		−0.61	−0.51	−0.54	

*, partial correlations (partial *r*) are estimated after removing effects of age and gender; life change, change of life habits due to quarantine; general disease, general level of disease caused by lockdown; BFO, Big Five Observer (E, extraversion; A, Agreeableness; C, Conscientiousness; S, Emotional Stability; O, Openness); LOT-R, Life Orientation Test Revised; BDI, Beck Depression Inventory; STAI, State-Trait Anxiety Inventory; HHI, Hope Herth Index (temp, temporality; conn, connectedness; exp, expentancy); MC, Marlowe-Crowne.

Cronbach’s α values indicate acceptable or good reliability for each psychological scale (Kline, 2000). There are significant correlations between the MC scale for social desirability and the psychological measures, but correlations have small effect sizes because they are lower than 0.5 (Cohen, 1992). Anxiety, assessed by STAI, and depression, assessed by BDI, are strongly correlated with each other (*r* = 0.65, *p* < 0.001) and are all significantly and negatively correlated with the five traits of personality (extraversion, agreeableness, conscientiousness, emotional stability, and openness). Correlations vary from −0.68 to −0.19 for STAI and from −0.59 to −0.25 for BDI. We also reported the partial correlations after removing the variance due to age and gender. The *p*-values of correlations were adjusted according to the false discovery rate procedure (Benjamini and Hochberg, 2000). Anxiety and depression have the highest correlations with emotional stability and the lowest correlations with openness. Therefore, personality traits have some relations on psychological well-being. Anxiety and depression are negatively correlated with optimism, assessed with LOT-R, and with the three dimensions of the HHI scale. Therefore, people with a high level of optimism and hopefulness are less affected by anxiety and depression. The variation of life habits (life change) has some relations on anxiety (*r* = 0.19, *p* < 0.001), but not on depression, whereas the disease caused by the lockdown is related to both o anxiety and depression (*r* = 0.42 and 0.36, respectively).

**FIGURE 5:**
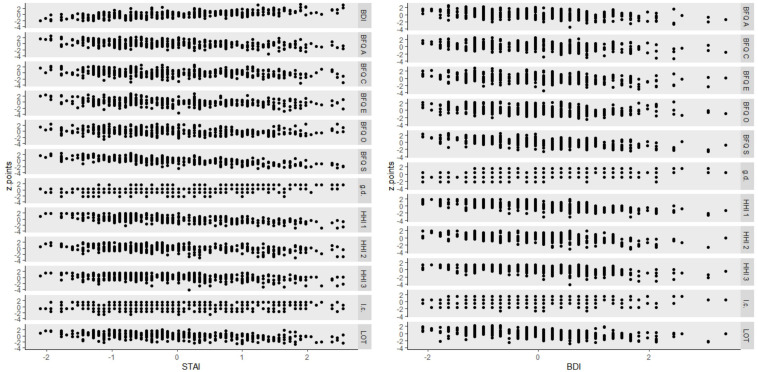
Scatterplots of the psychological measures (I.c., life change; g.d., general disease; BFQ E, extraversion; BFQ A, agreeableness; BFQ C, conscientiousness; BFQ S, emotional stability; BFQ O, openness; HHI1, temporality subscale of hopefulness; HHI 2, interconnectedness subscale of hopefulness; HHI 3, positive expectancy subscale of hopefulness) in relation to anxiety (STAI) and depression (BDI). Raw scores were transformed into standardized scores (z points) for allowing comparison between scales.

Figure 5 shows the scatterplots of the correlations reported in Table 5, in relation to anxiety, assessed with the STAI, and depression, assessed with the BDI. Raw scores were transformed into standardized scores (z points) for allowing comparisons between scales. Scatterplots do not evidence particular anomalies in data distributions.

**FIGURE 6:**
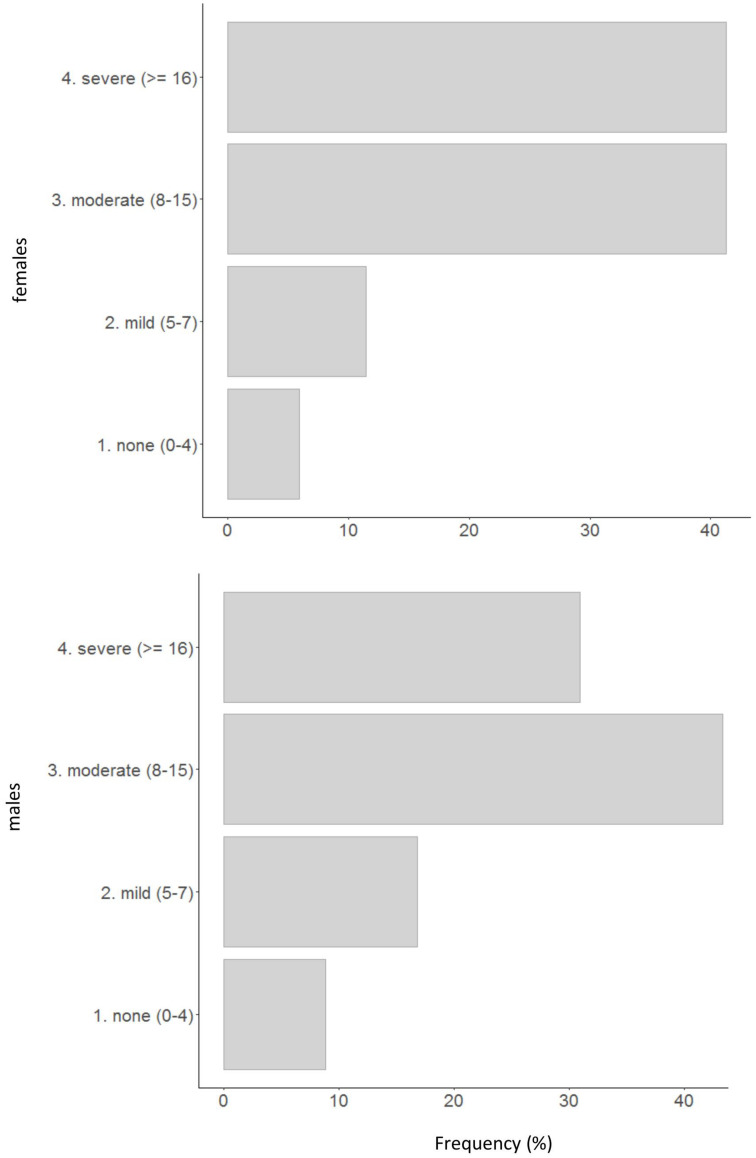
Frequency of depression levels in relation to BDI scores.

Of the participants, 15.04% of males obtained scores at the STAI equivalent to or higher than 55 (95th percentile for the male population), whereas 16.06% of females obtained scores at the STAI equivalent to or higher than 61 (95th percentile for the female population). Thus, the level of anxiety in the Italian sample during the phase one lockdown is practically tripled in relation to the cutoffs estimated in the normal population in the prepandemic period (Sanavio, 1997). The values of STAI corresponding to the mean of the Italian population are 37 for males and 40 for females (Sanavio, 1997). During the lockdown, the mean values of STAI increased to 41.45 and 47.38 for males and females, respectively. Therefore, the presence of a higher level of anxiety during the lockdown is confirmed by empirical data. Figure 6 shows the frequencies of the level of depression (none, mild, moderate, and severe) in participants. More than 50% of participants show moderate or severe symptoms of depression. In particular, 41.31% of females and 30.97% of males show severe symptoms of depression, respectively. Therefore, the presence of a high level of depression during the lockdown is also confirmed.

## Discussion

During the lockdown period, people are obliged to stay at home, even if they have permission to go out (to buy food, medicines, or for strong necessities). The data collected from our sample show that people, during their mandatory permanence at home, are not totally inactive, and that they follow the provisions established by the Ministry of Public Health to avoid contamination (use of masks and glove, washing hands, safety distance observance from others, permanence at home). Only very few people declared to have been contaminated by COVID-19, and many of them still claimed to be in a good health conditions, even when they were people suffering from other chronic diseases. About 20% of individuals reported having at least one relative or friend infected by COVID-19, and this could have affected their psychological status. The physical symptoms reported by participants were, above all, symptoms connected with a stressful condition of life. Migraine, sleep disorders, difficulty of concentration, and persistent exhaustion are typical signs of stress (American Psychological Association, 2010). Participants were clearly aware that COVID-19 was not a banal flu, that it could contaminate everyone, and that contamination could happen through air dispersion of the virus or through contact with contaminated objects. They were worried about a possible contamination toward themselves or toward their relatives or friends, about a possible virus mutation, or about the difficulty to find a valid therapy against the virus.

The principal result of our study is that the level of anxiety and the level of depression are very high in Italians during the lockdown period. The percentage of extreme anxiety is tripled, in relation to the prepandemic measured levels (Sanavio, 1997), and about 50% of participants show a moderate or severe level of depression. About 30% of males and 41% of females suffer from severe depression. Therefore, there are evidences that the lockdown have had negative effects on psychological well-being. However, some participants showed low levels of anxiety and depression, in particular, those who had high level of optimism and hopefulness. These people have a positive vision of the future and a strong confidence that their actions and behaviors can improve the negative situation and these characteristics, reasonably, can attenuate their psychological sufferance. Also, personality traits have some relations with anxiety and depression. People with high level of extraversion, agreeableness, conscientiousness, emotional stability, and openness have lower tendency to suffer from anxiety and depression.

Generally, the negative effects of the lockdown on psychological conditions are evident, confirming the importance of mental health prevention (Marazziti et al., 2020) and the necessity of psychological interventions against the negative impact of the pandemic on individuals (Holmes et al., 2020). Participants need psychological support, even if they do not suffer from mental or physical diseases (Brooks et al., 2020; Marazziti et al., 2020). Because of the impossibility to freely move outside the home during the lockdown, it could be useful to develop digital technologies for providing psychological support *via* internet, social networks, or apps for smartphones (McCord et al., 2015; Silva et al., 2015; Orrù et al., 2020). Through internet, it could be possible not only to provide direct assistance to people at home with the help of clinical psychologists and psychiatrists but also to teach and explain techniques for reducing psychological diseases and improving well-being, as, for example, mindfulness (Bullis et al., 2014; Saggino et al., 2017; Sood, 2020).

Both governments and private institutions should invest on mental health care of citizens, when big catastrophes happen. When negative events with a radical impact on population activities happen, remedies for the population should be taken to overcome the consequential diseases (Holmes et al., 2020; Saglietto et al., 2020). Psychological diseases should not be neglected, because they have negative consequences on individuals, institutions, societies, and governments (Gyani et al., 2013; Layard, 2013; Brooks et al., 2020).

In 4 May, the Italian government declared the passage to the phase two of the pandemic for 18 May. Limits and restrictions of movement for the population were reduced, and some economic activities could restart. To test the evolution of anxiety and depression in this new phase, we asked participants if they wanted to repeat the survey. About 60% accepted positively to repeat the test.

One possible limit of this research is that the online survey cannot guarantee a perfect randomized selection of participants, but this was the only possibility because of the limits imposed by the lockdown. However, the different channels used to propagate the survey and the high number of participants allowed a collection of data from a sample composed of heterogeneous individuals of different parts of Italy. Another possible limit is the use of psychological tests, especially for the estimation of depression, that are not the typical test used by professional psychiatrists or clinical psychologists. However, we have to say that the BDI was used in more than 2000 studies (Richter et al., 1998), and that it is widely used by Italian clinical psychologists (Sica and Ghisi, 2007).

## Data Availability Statement

The data analyzed in this study are subject to the following licenses/restrictions: Datasets are conserved by the authors in a safe place in respect of the Italian laws for the privacy. Requests to access these datasets should be directed to MT, marco.tommasi@unich.it.

## Ethics Statement

Ethical review and approval was not required for the study on human participants in accordance with the local legislation and institutional requirements. The patients/participants provided their written informed consent to participate in this study.

## Author Contributions

MT contributed to providing materials, data analysis, programming online survey, and searching of literature sources. FT contributed to collecting participants, providing materials, helping in manuscript revision, and searching of literature sources. SA contributed to collecting participants, programming online survey, and helping in manuscript revision. AC, MC, MD, and MS contributed to collecting participants, helping in manuscript revision, and searching of literature sources. LP and AS contributed to data analysis, helping in manuscript revision, and searching of literature sources. All authors contributed to the article and approved the submitted version.

## Conflict of Interest

The authors declare that the research was conducted in the absence of any commercial or financial relationships that could be construed as a potential conflict of interest.

## Footnotes

1 https://gisanddata.maps.arcgis.com
